# Factors Contributing to Tumor Shrinkage after Peptide Receptor Radionuclide Therapy in Patients with Unresectable Neuroendocrine Tumors

**DOI:** 10.3390/cancers14143317

**Published:** 2022-07-07

**Authors:** Sho Hasegawa, Noritoshi Kobayashi, Damian Wild, Fesupplix Kaul, Naoki Okubo, Akihiro Suzuki, Yusuke Kurita, Shoko Takano, Atsushi Nakajima, Yasushi Ichikawa

**Affiliations:** 1 Gastroenterology and Hepatology Division, Yokohama City University Hospital, Yokohama 2360004, Japan; t166064d@yokohama-cu.ac.jp (S.H.); kuritay@yokohama-cu.ac.jp (Y.K.); nakajima-tky@umin.ac.jp (A.N.); 2 Oncology Division, Yokohama City University Hospital, Yokohama 2360004, Japan; t206009g@yokohama-cu.ac.jp (N.O.); aksuzuki@yokohama-cu.ac.jp (A.S.); yasu0514@yokohama-cu.ac.jp (Y.I.); 3 Nuclear Medicine Division, University Hospital Basel, 4031 Basel, Switzerland; damian.wild@usb.ch (D.W.); felix.kaul@usb.ch (F.K.); 4 Radiation Oncology Division, Yokohama City University Hospital, Yokohama 2360004, Japan; tshoko@yokohama-cu.ac.jp

**Keywords:** lesion-based analysis, neuroendocrine tumors, peptide receptor radionuclide therapy

## Abstract

**Simple Summary:**

Neuroendocrine tumors (NETs) are rare disorders of neuroendocrine cells with an increasing incidence. Surgical resection is the only cure; however, they are often found at an advanced stage, and many cases are unresectable because of being locally advanced or presenting as metastatic disease. Peptide receptor activation therapy (PRRT) has been found to be effective for metastatic NETs. Prediction of tumor shrinkage after PRRT for metastatic NETs is challenging and remains unclear. This study aimed to identify predictive factors associated with the rate of PRRT tumor shrinkage. This study performed both patient-based and lesion-based shrinkage analysis for metastatic NETs. We analyzed the relationship between pretreatment clinicopathological factors and the shrinkage rate per lesion (L-SR) in 20 patients. Previous treatment with cytotoxic agents and primary tumor of the pancreas were found to be significantly favorable factors; however, a primary tumor of the rectum was significantly more resistant to shrinkage.

**Abstract:**

Peptide receptor activation therapy (PRRT) is a promising treatment option for metastatic neuroendocrine tumors (NETs). However, predicting tumor shrinkage before treatment is challenging. We analyzed the shrinkage rate of each metastatic tumor lesion to identify predictive factors related to shrinkage. Patients with metastatic NET who underwent PRRT were included in this retrospective study. For each patient, between one to five metastatic lesions were selected in descending order of size, and the change in the maximum tumor diameter after treatment was defined as the shrinkage rate per lesion (L-SR). We analyzed the relationship between pretreatment clinicopathological factors and L-SR. The median L-SR of all 75 lesions in 20 patients was 20% (95% CI: 4.8–26.1%). While previous treatment with cytotoxic agents (34.4%, *p* < 0.05) and primary tumor of the pancreas (27.8%, *p* < 0.05) were significantly favorable factors, a primary tumor of the rectum was significantly more resistant to shrinkage (−20.5%, *p* < 0.001). Therefore, lesion-based analysis of PRRT for NETs showed that pancreatic NET and previous treatment with cytotoxic agents were favorable factors for tumor shrinkage; however, rectal NET was a factor associated with resistance to shrinkage.

## 1. Introduction

Neuroendocrine tumors (NETs) are rare disorders of neuroendocrine cells that progress relatively slow, but their incidence is increasing [1,2]. Although surgical resection is the only curative treatment for NETs [3,4], nonfunctional NETs are often found at an advanced stage, and many cases are unresectable because of being locally advanced or presenting as metastatic disease. Many systemic treatments have been recently introduced for NETs, including somatostatin analogs, cytotoxic agents, and targeted molecular therapies. Although somatostatin analogs and targeted molecular therapies show promise in terms of progression-free survival (PFS), the response rate (RR) of sunitinib was 9.3% and that of everolimus was 4%, neither of which is high [5,6,7]. The RR of everolimus for pancreatic NETs (pan NETs) was 5%, and that for lung and gastrointestinal NETs was 2%. Targeted molecular therapy may also occasionally lead to relatively severe adverse events, and long-term continuation of therapy may worsen quality of life. In treatments with cytotoxic agents, the RR of streptozotocin-based chemotherapy is approximately 33–40% and that of temozolomide-based chemotherapy is 27.8–33.3% [8,9,10]; however, long-term continuation of therapy with these agents is difficult because of the moderate hematological and non-hematological damage that often results from such therapy.

In the NETTER-1 study, peptide receptor radionuclide therapy (PRRT) was shown to significantly prolong PFS (28.4 months vs. 8.4 months) and had a relatively high RR in comparison with high-dose octreotide therapy (18% vs. 3%) [11]. The RR of PRRT has been reported to be approximately 29–57% for pan NETs, and 17–73% for gastroenteropancreatic NET. Thus, PRRT has been shown to be a more reliable treatment option for tumor shrinkage than other systemic treatments.

Somatostatin receptor scintigraphy (SRS) is one of the most important and necessary modalities for determining PRRT adaptation. A retrospective study in 2008 revealed that the uptake score on pretreatment SRS was a prognostic factor for predicting tumor remission [12]. However, the RR in that study was only 60%, even with an intense uptake of ^111^In-pentetreotide; in contrast, approximately 40% of cases could not achieve tumor shrinkage rates over 30%. Another retrospective study revealed that the ^68^Ga-DOTATOC maximum standardized uptake value (SUV_max_) cutoff of 16.5 showed a 95% sensitivity and 60% specificity for response prediction [13]. This low specificity indicated that predictive tumor shrinkage was not sufficient, even by pretreatment ^68^Ga-DOTATOC analysis.

Many other clinicopathological factors for predicting tumor shrinkage have been investigated, including tumor diameter, primary lesion, and pathological grade. More recent findings from the NETTER-1 study showed that the target lesions were divided into two groups based on tumor diameter, ≤30 mm and >30 mm [14]. The least squares mean shrinkage was 29% and 14%. There was a significant interaction of baseline tumor size and liver tumor size shrinkage. According to another report focused on tumor origin, tumors in the small intestine have a lower RR compared with particular pan NETs [15].

Other studies evaluated the RR of PRRT by pathological grade [11,16]. The NETTER-1 study showed no value of grade 1 versus grade 2 tumors for predicting response to PRRT. Sorbye et al. described PRRT in grade 3 as achieving high RR (31–41%); however, PFS was lower than in other studies for grade 1 and grade 2 NETs [16].

In the past, many studies evaluated tumor shrinkage by RR according to the Response Evaluation Criteria in Solid Tumors guideline version 1.1 (RECIST) criteria; in other words, very few reports have described both patient-based and lesion-based analysis.

Prediction of tumor shrinkage after PRRT for metastatic NETs is challenging and needs to be better understood; accurate prediction could prove valuable to clinicians seeking to optimize treatment and determine prognosis. Therefore, this study aimed to identify predictive factors associated with the rate of PRRT tumor shrinkage. In this study, we performed both patient-based and lesion-based shrinkage analysis for metastatic NETs.

## 2. Materials and Methods

### 2.1. Patients

We retrospectively evaluated patients aged ≥20 years who were referred from the Yokohama City University Hospital (Yokohama, Japan) to the University Hospital Basel (Basel, Switzerland) for PRRT between 2011 and 2020 [17]. The diagnosis of NET was made by pathological tissue retrieval following the World Health Organization (WHO) 2019 classification [18]. The patients had a varied history of treatment before PRRT, including surgical treatment, somatostatin analog treatment, targeted molecular therapies, cytotoxic agent therapy, and topical therapy (transarterial chemoembolization and radionics wave ablation). Somatostatin analogs were discontinued six weeks prior to PRRT in patients who were still receiving them. This study was approved by the Ethics Committee of Yokohama City University (F210800004). Informed consent was acquired from all participants using an opt-out method.

### 2.2. PRRT Protocol

DOTATOC was synthesized according to a five-step procedure in accordance with good laboratory practices at the University of Basel [19]. ^90^Y-DOTATOC and ^177^Lu-DOTATOC were alternately used until December 2017, and ^177^Lu -DOTATOC single-agent treatment was used thereafter. Radiolabeling was performed using 3.7 GBq/m^2^ body surface of ^90^Y, a beta emitter, for therapeutic purposes, and 0.111 GBq of ^111^Indium, a gamma emitter, for internal imaging for therapeutic purposes. To inhibit tubular reabsorption of radiopeptides, an intravenous infusion of 1000 mL of saline containing 20.7 mg/mL of arginine and 20.0 mg/mL of lysine was started 30 min before the ^90^Y-DOTATOC injection and continued until 3 h later. The patients were hospitalized for 3 d per cycle in accordance with the Swiss requirements for legal radiation protection. Considering the patient burden associated with travel from Japan to Basel, three cycles of PRRT were performed in one treatment.

### 2.3. Somatostatin Receptor Imaging

The distribution of radiopeptides during the treatment was acquired using a dual-head hybrid single-photon emission computed tomography (SPECT)/computed tomography (CT) gamma camera combined with a SPECT unit and 16-slice CT unit, the Symbia T16 (Siemens Healthcare, Hoffman Estates, IL, USA) system. Planar images of the whole body were acquired 4 and 24 h after intravenous injection of 148–222 MBq of ^111^In-pentetreotide. Images were visually analyzed and abnormal SRS findings were defined as increased non-physiological uptake. The maximum grade was scored according to the Krenning scale on a five-point scale from 0 to 4 [20].

### 2.4. Image Analysis Method

All CT scans were performed using a multidetector helical CT system (Aquilion; Toshiba Medical Systems, Tokyo, Japan) with 16 or 64 detector rows, a spin time of 0.5 s, a radiation exposure dose coefficient of 120 kV, and 250–300 mAs. For post-treatment lesion evaluation, CT was performed approximately 10–12 weeks after completion of the three PRRT cycles. Lesions were selected from those with a score of 2 or higher on SRS, and 1–5 lesions were evaluated per patient. The longest diameter of the target lesion was measured by CT and compared before and after PRRT treatment to calculate the shrinkage rate. The growth rate was measured from the tumor size before and after PRRT, and the tumor doubling time was measured from the two-point CT imaging before PRRT.

### 2.5. Outcome Measurements

The primary outcome of this study was the median best shrinkage rate per lesion (L-SR) after PRRT. The secondary outcomes were the results obtained by analysis of factors associated with the best shrinkage rate. The primary lesion site; Krenning scale; Ki-67 labeling index (LI); previous treatment (cytotoxic anticancer agents, molecular targeted agents, somatostatin analogs); doubling time; and tumor size were evaluated as relevant factors. Tumor diameters were measured using up to five lesions (up to two lesions in each organ) as target lesions, based on RECIST, and the rate of change in the sum of tumor diameters before and after PRRT was determined as the shrinkage rate per patient (P-SR). The factors associated with P-SR were analyzed in the same manner for each lesion. In addition, the long-term course of P-SR, including the appearance of new lesions, was evaluated using RECIST.

### 2.6. Statistical Analysis

Continuous variables were compared using the Mann–Whitney U test or *t*-test, while categorical variables were compared using the chi-square test or Fisher’s exact test. A *p* value < 0.05 was considered significant. A logistic regression model was used for the multivariate analysis. Variables with *p* < 0.05 in the univariate analysis were entered into the multivariate analysis. Statistical analyses were performed using the JMP15.0 statistical software (SAS Institute Inc., Cary, NC, USA).

## 3. Results

### 3.1. Patient Characteristics

Twenty patients with NET (seven men; thirteen women) underwent SRS followed by PRRT, after excluding those patients who did not undergo SRS and those who could not undergo three sessions of PRRT. The median age at the time of the first PRRT session was 59 years (range: 39–68 years). The primary tumor sites were the pancreas (10 patients), rectum (5 patients), stomach (1 patient), small intestine (1 patient), thymus (1 patient), bile duct (1 patient), or unknown (1 patient). Grade 1 and 2 tumors according to WHO classification 2019 were reported in 3 and 17 patients, respectively; no grade 3 tumors were identified. The maximum accumulation score of SRS per case based on the Krenning scale was 0, 1 in no patients, 2 in 1 patient, 3 in 1 patient, and 4 in 18 patients. In the lesion-based analysis, the Krenning scale was 0, 1 for no lesions, 2 for 3 lesions, 3 for 10 lesions, and 4 for 62 lesions. Previous treatment comprised surgical resection in 16 patients, targeted molecular therapies in 11 patients, cytotoxic agent therapy in 5 patients, and somatostatin analog treatment in 16 patients (Table 1).

### 3.2. Factors Associated with Best tumor Shrinkage Rate in Lesion-Based Analysis

**Figure 1:**
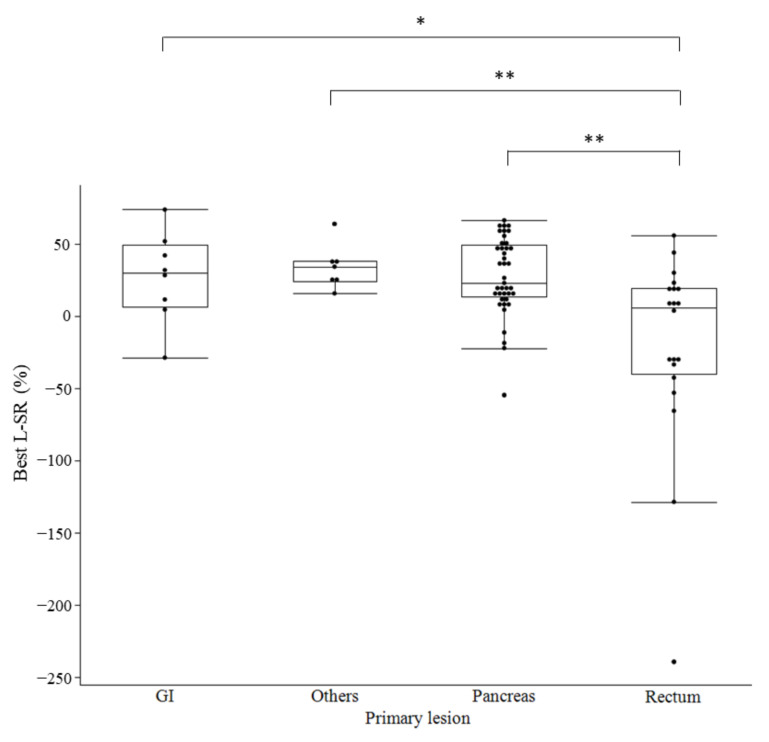
Box-and-whisker plot of lesion-based shrinkage rate for each organ. L-SR, Lesion-based shrinkage rate; GI, gastrointestinal tract; * *p* < 0.05, ** *p* < 0.01.

In total, 75 lesions with SRS scores of 2 or higher in 20 patients were evaluated for shrinkage and the median best L-SR was 20% (95% CI: 4.8–26.1%). The median time of best L-SR was 37.5 weeks (range: 19–231 weeks) after PRRT. The number of lesions and best L-SR are shown in Table 2, and no significant difference was noted in relation to the Krenning scale (score 2: 15.8%, 95% CI (−77.4% to 88.5%); score 3: 3.8% (−35.4% to 43.2%); score 4: 17.8% (6.3–29.4%)). In comparisons based on the primary lesion site, the best L-SR was significantly higher for pancreatic lesions (27.8% (19.2–36.3%) *p* = 0.0295) and significantly lower for rectal lesions (−20.5% (−51.9% to 11.0%) *p* = 0.0002). The box-and-whisker plot of L-SR by organ showed that PRRT was less effective in the rectum than in the pancreas and that there was greater variability among lesions (Figure 1). When the Ki-67 LI was compared with 10% as the cutoff, no significant difference was observed in the L-SR (Ki-67 LI < 10%: 22.9% (14.8–31.0%); Ki-67 LI > 10%: −2.48% (−33.5% to 29.5%); *p* = 0.1984). When doubling time was compared with 100 days as the cutoff time, no significant difference was observed in the L-SR (<100 days: 6.1% (−11.5% to 23.7%); >100 days: 28.1% (21.1–35.0%); *p* = 0.1141).

In comparisons based on the radionuclides used in the treatment, no significant difference was observed in the L-SR (^90^Y and ^177^Lu combination: 10.6% (−9.5% to 30.6%); ^177^Lu monotherapy: 19.5% (8.7–30.3%); *p* = 0.7095). No significant difference was observed in the L-SR between patients with and without a history of use of targeted molecular therapy (with use: 22.6% (14.3–31.1%); without use: 2.66% (−23.2% to 28.6%); *p* = 0.2943) or somatostatin analog therapy (with use: 11.4% (−1.1% to 23.9%); without use: 31.7% (13.3–50.2%); *p* = 0.0523), but the L-SR was significantly higher in patients with a history of cytotoxic agent use (with use: 25.2%; without use: 19.5%; *p* = 0.0394).

The L-SR showed no difference comparing patients with and without somatostatin analog (SSA) maintenance treatment after PRRT (with SSA: 2.1% (−28.3% to 32.4%); without SSA: 21.0% (12.2–29.9%); *p* = 0.4887). There was no difference in the L-SR on comparing pretreatment tumor diameters with a cutoff of 3 cm (13.8% (0.29–27.3%), >3 cm: 20.7% (7.57–33.9%), *p* = 0.9062).

Seventy-five tumor lesions were classified into two groups by the median value for the L-SR (20%), and clinicopathological factors associated with L-SR were analyzed using univariate and multivariate analyses. (Table 3). In univariate analysis, the factor with a significantly negative impact on tumor L-SR was rectal primary tumor (*p* = 0.0041). In multivariate analysis with rectal primary tumor, other primary tumor, history of somatostatin analog use, and tumor size, only rectal primary tumor had a significantly negative impact on the L-SR of PRRT (odds ratio, 0.21; 95% CI (0.05–0.76); *p* = 0.0184).

### 3.3. Per-Patient Analysis of the Change in Tumor Size over Time Based on RECIST

**Figure 2:**
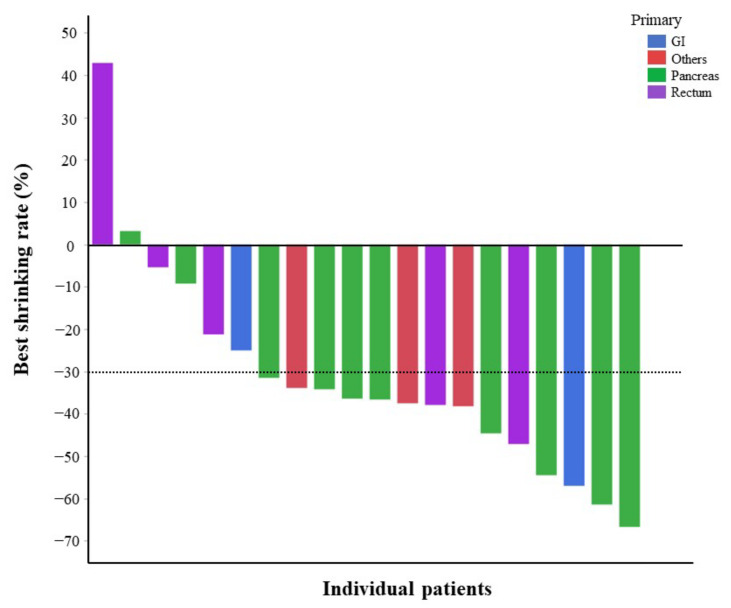
Waterfall plot showing the best shrinkage rate by PRRT in patient-based analysis. Green line, primary site of pancreas; blue line, primary site of gastrointestinal tract; purple line, primary site of rectum; red line, primary site of others. PRRT, peptide receptor activation therapy.

Based on RECIST, one to five lesions (up to two lesions in each organ) were selected as target lesions in each patient in the order of tumor size, and the rate of change in the sum of tumor diameters was defined as the P-SR for each patient. The best PRRT P-SR results are presented in Table S1. None of the factors were significantly related to the best P-SR in analyses with the Krenning scale, primary tumor site, Ki-67 LI, radiotherapeutic agents, prior therapy, SSA maintenance therapy, and tumor diameter. When the best P-SR was presented in a waterfall plot by primary site (Figure 2), primary pancreatic lesions tended to show more shrinkage than other primary lesions.

**Figure 3:**
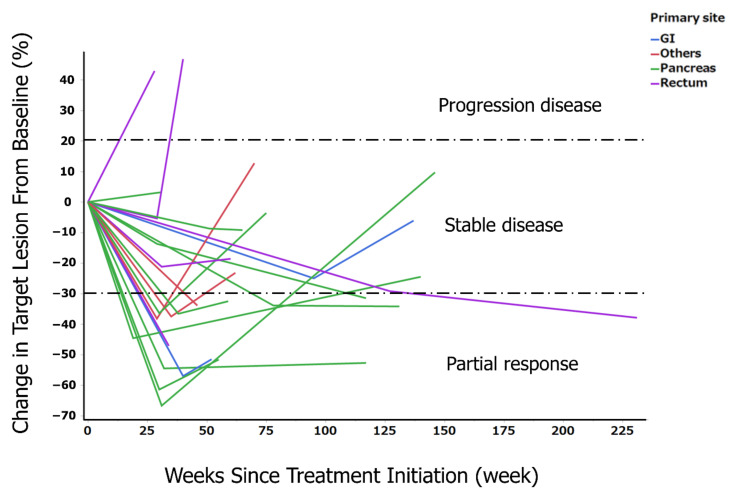
Spider plot showing the change in sum of tumor diameters based on RECIST in the 20 patients. RECIST, Response Evaluation Criteria in Solid Tumors guideline version 1.1; green line, primary site of pancreas; blue line, primary site of gastrointestinal tract; purple line, primary site of rectum; red line, primary site of others.

The long-term course of P-SR was examined in each patient. The rate of change from the baseline was clearly more sustained in the patients with primary pancreatic tumors than in those with a primary rectal tumor (Figure 3). The long-term response rate of PRRT was 40% (patients with CR0, PR8, SD11, PD1) at 25 weeks after PRRT; 35% (patients with CR0, PR7, SD7, PD4, two unevaluable patients) at 50 weeks after PRRT; and 20% (patients with CR0, PR4, SD3, PD9, four unevaluable patients) at 100 weeks after PRRT (Table 4).

## 4. Discussion

In this lesion-based analysis of the efficacy of PRRT, the median rate of maximum L-SR was 20% and the time of maximum shrinkage was 37.5 weeks (range: 19–231 weeks) after PRRT. Multivariate analysis showed that the location of the primary tumor in the rectum was a poor response factor. In the patient-based analysis, no factors were significantly associated with P-SR. The novelty of this study is that the shrinkage rate of PRRT differs for each lesion. In addition, the shrinkage rate may differ depending on the primary site. It may be possible to predict treatment efficacy, including the primary site and the time of maximum shrinkage, when conversion surgery using PRRT is considered.

The RR to PRRT was relatively high (26–56%) in previous reports [21,22,23,24,25]. In the present study, the RR per patient was 40% (at 25 weeks), which is similar to findings of previous reports on PRRT. Previous research has also examined RR per primary organ, with Brabander et al. describing an RR of 55% for pan NET, 31% for midgut NET, and 33% for hindgut NET [25].

In the present study, when the PRRT reduction rate was evaluated according to the primary organ, a favorable trend in pan NET was observed, while rectal NET was found to be a significantly poor factor. One of the reasons for this may be that the L-SR of the patients with primary rectal tumors in this study varied greatly across lesions and was more heterogeneous than in other organs according to the box-and-whisker plot. In addition, the pancreas originates from the foregut and the rectum from the hindgut, and the different origins of the organs may have influenced the results of different tumor shrinkage. In fact, the response rate of PRRT for midgut NET (18%) is lower than that of other primary lesions (26–56%) [11,21,22,23,24,25]. Primary lesions may be one of the most reliable predictive factors of tumor shrinkage; however, there may be other factors related to the response of PRRT that have not yet been identified. There was no significant difference in the duration of disease among organs (detailed data not shown) in this study, although the overall duration of disease before PRRT was long and may have been greatly influenced by previous treatment.

The efficacy of PRRT was retrospectively analyzed in 27 patients with rectal NET in a previously reported series [26]. Partial response was observed in 70% of the patients; the median PFS was 29 months; and the median overall survival (OS) was 81 months. In this study, a high morphological response and long PFS and OS were achieved in patients with metastatic rectal NETs. However, 15 patients (51.9%) received PRRT with radio sensitizer (5-FU or capecitabine), and one sigmoid NET was included in this study. According to another study on the efficacy of PRRT in 12 rectal NETs, the partial RR was 33%, with a median PFS of 29 months [25]. Another study reported that the overall RR was only 15.7% in hindgut NETs, including rectal NETs [27]. In general, rectal NETs with distant metastasis have a poor prognosis with a median OS of 4–11 months [2,28]. The efficacy of PRRT for rectal NETs remains controversial because the number of reported cases is very small, including our data. Therefore, we believe that the accumulation of more cases of rectal NETs is necessary for future research.

Uptake of SRS before PRRT is one of the most important significant prognostic factors for predicting tumor remission [12]. However, in the present study, there was no significant correlation between SRS score and tumor shrinkage. The most important reason for this finding was the insufficient number of lesions to analyze the correlation between SRS score and tumor shrinkage. Nevertheless, the SRS score alone cannot provide adequate information for predicting treatment response. Ga-DOTATOC-PET is a more reliable imaging modality for quantity analysis and prediction of tumor response than SRS [13].

Regarding the presence or absence of large lesions (>3 cm), a significant difference in PFS was reported in the NETTER-1 study [15]. However, the present study showed no difference in tumor L-SR based on the presence or absence of large lesions (>3 cm). In this study, half of all patients received ^90^Y and ^177^Lu combination PRRT; therefore, ^90^Y may be more effective for large lesions. The combination of ^90^Y and ^177^Lu has been reported as being more effective than ^177^Lu monotherapy [15].

In the lesion-based analysis, previous treatment with cytotoxic agents was favorable for the therapeutic effect of PRRT. No difference in PRRT efficacy related to prior treatment has been reported in previous studies. The reason for this discrepancy was that the median time from diagnosis to PRRT in this study was 61.5 months, which was much longer than that of previously reported studies. It is possible that cytotoxic agent usage was extracted as a significant factor as a result of the implementation of various multidisciplinary therapies for NETs in comparison with other reports.

In recent years, there have been scattered reports of the usefulness of PRRT as neoadjuvant therapy for locally advanced unresectable NETs. Parghane et al. reported that after ^177^Lu-DOTATATE therapy, the unresectable primary tumor became resectable in 15 of 57 (26.3%) patients [29]. PRRT may be considered not only as a palliative but also as a neoadjuvant therapy; therefore, the prediction of tumor shrinkage before PRRT is a meaningful evaluation that may lead to curative treatment. Although further accumulation of cases is essential, the primary tumor site and previous treatment history may provide some guidance in this regard.

This study had several limitations. First, this was a single-center retrospective study with a small number of patients. Second, the treatment comprised three PRRT sessions, fewer than the 4–6 sessions reported previously. Third, pathological factors were not always evaluated in the target lesions, and Ki-67 LI values included the results for some patients without liver metastases. Furthermore, the evaluation time of the images varied from case to case, making uniform evaluation difficult.

## 5. Conclusions

Despite the limitations, this is a valuable report evaluating the lesion-based therapeutic efficacy of PRRT in patients with NETs. In this study, pan NET and previous cytotoxic anticancer drug use were good predictors of response to PRRT, while rectal NET was a poor predictor.

## Figures and Tables

**Table 1:** Patient characteristics.

	*n*
Patients	20
Sex	
Male	7
Female	13
Age, median, (range)	59 (39–68)
Primary site	
Pancreas	10
Rectum	5
Stomach	1
Small intestine	1
Thymus	1
Biliary tract	1
Unknown	1
WHO classification	
G1	3
G2	17
G3	0
SRS score	
Score 0, 1	0
Score 2	1
Score 3	1
Score 4	18
Previous treatment	
Surgical operation	16
Cytotoxic agents	5
Targeted molecular therapies	11
Somatostatin analog	16
PS	
0–1	20
2–4	0
Period from diagnosis to PRRT, month, (range)	61.5 (5–186)
Hereditary status	
Non-hereditary	19
MEN type 1	1

WHO, world health organization; SRS, somatostatin receptor scintigraphy; PS, performance status; PRRT, peptide receptor radionuclide therapy; MEN, multiple endocrine neoplasia.

**Table 2:** Factors associated with tumor shrinkage in NET in lesion-based analysis.

Variable		Number of Lesions, *n* (%)	Shrinkage Rate, % (Median [95%CI])	*p* Value
All lesion		75 (100)	20 [4.8~26.1]	
SRS score	score 2	3 (4)	15.8 [−77.4~88.5]	0.3944
	score 3	10 (13.3)	3.8 [−35.4~43.2]	0.4782
	score 4	62 (82.7)	17.8 [6.3~29.4]	0.2749
Primary lesion	Pancreas	40 (53.3)	27.8 [19.2~36.3]	0.0295
	Rectum	20 (26.7)	−20.5 [−51.9~11.0]	0.0002
	GI	8 (10.7)	27.1 [0.77~53.3]	0.5480
	Others	7 (9.3)	34.4 [20.3~48.6]	0.1749
Ki67 labeling index	<10%	53 (70.7)	22.9 [14.8~31.0]	0.1984
	>10%	22 (29.3)	−2.48 [−33.5~29.5]	
Doubling time	<100 days	43 (57.3)	6.1 [−11.5~23.7]	0.1141
	>100 days	32 (42.7)	28.1 [21.1~35.0]	
Therapeutic drug	^90^Y and ^177^Lu-DOTATOC	34 (45.3)	10.6 [−9.5~30.6]	0.7095
	^177^Lu-DOTATOC	41 (54.7)	19.5 [8.7~30.3]	
Previous treatment				
Cytotoxic agent	yes	18 (24)	34.4 [−23.6~45.2]	0.0394
	no	57 (76)	9.49 [−3.8~22.8]	
Target molecular therapy	yes	48 (64)	22.6 [14.3~31.1]	0.2943
	no	27 (36)	2.66 [−23.2~28.6]	
Somatostatin analog	yes	60 (80)	11.4 [−1.1~23.9]	0.0523
	no	15 (20)	31.7 [13.3~50.2]	
SSA maintenance treatment	yes	22 (29.3)	2.1 [−28.3~32.4]	0.4887
	no	53 (70.7)	21.0 [12.2~29.9]	
Tumor size before PRRT	<3.0 cm	57 (76)	13.8 [0.29~27.3]	0.9062
	>3.0 cm	18 (24)	20.7 [7.57~33.9]	

NET, neuroendocrine tumor; SRS, somatostatin receptor scintigraphy; CI, confidence interval; GI, gastrointestinal tract; SSA, somatostatin analog; PRRT, peptide receptor radionuclide therapy.

**Table 3:** Univariate and multivariate analysis of factors affecting tumor shrinking effect of PRRT in lesion-based analysis.

Factor		*n*	Effectiveness (%)	Univariate *p*	Multivariate *p*	OR	95% CI
Primary	Pancreas	40	52.5	0.4893			
	Rectum	20	20	0.0041	0.0184	0.21	0.05~0.76
	GI	8	62.5	0.4692			
	Others	7	85.7	0.0502	0.1466	0.19	0.02~1.78
SRS score	Score 2	3	33.3	1.000			
	Score 3	10	50	1.000			
	Score 4	62	48.4	1.000			
Ki67 LI	<10%	53	50.9	0.4583			
	>10%	22	40.9				
Doubling time	>100 days	32	44.2	0.4897			
	<100 days	43	53.1				
Therapeutic drug	^90^Y and ^177^Lu DOTATOC	34	44.1	0.6440			
	^177^Lu DOTATOC	41	51.2				
Previous treatment							
Cytotoxic agent	Yes	57	45.6	0.5901			
	No	18	55.7				
Targeted molecular agent	Yes	48	50.0	0.8101			
	No	27	44.4				
Somatostatin analog	Yes	60	43.3	0.1497	0.0736	0.30	0.89~1.12
	No	15	66.7				
SSA maintenance treatment	Yes	22	50	1.000			
	No	53	47.1				
Tumor size before PRRT	<3.0 cm	57	52.6	0.1836	0.5044	0.65	0.19~2.24
	>3.0 cm	18	33.3				

SRS, somatostatin receptor scintigraphy; CI, confidence interval; GI, gastrointestinal tract; SSA, somatostatin analog; PRRT, peptide receptor radionuclide therapy.

**Table 4:** Long-term outcomes after PRRT based on RECIST in the 20 patients.

Response Criteria	25 Weeks	50 Weeks	100 Weeks
Response, *n* (%)	8 (40)	7 (35)	4 (20)
CR, *n* (%)	0 (0)	0 (0)	0 (0)
PR, *n* (%)	8 (40)	7 (35)	4 (20)
SD, *n* (%)	11 (55)	7 (35)	3 (15)
PD, *n* (%)	1 (5)	4 (20)	9 (45)
Not evaluated, *n* (%)	0 (0)	2 (10)	4 (20)

PRRT, peptide receptor radionuclide therapy; RECIST, Response Evaluation Criteria in Solid Tumors guideline version 1.1; CR, complete response; PR, partial response; SD, stable disease; PD, progression disease.

## Data Availability

The data presented in this study is available in this article (and Supplementary Materials).

## Supplementary Materials

The following supporting information can be downloaded at: https://www.mdpi.com/article/10.3390/cancers14143317/s1, Table S1: Factors associated with tumor shrinkage in NET in patient-based analysis.
