# Supplementary material for: Factors Contributing to Tumor Shrinkage after Peptide Receptor Radionuclide Therapy in Patients with Unresectable Neuroendocrine Tumors
Source: Cancers (Basel). 2022 Jul 7;14(14):3317. doi: 10.3390/cancers14143317 (PMC9313286; doi:10.3390/cancers14143317)
Supplement: Supplementary file 1 [file cancers-14-03317-s001.zip › cancers-1758590-supplementary.pdf]

Table S1. Factors associated with tumor shrinkage in NET in patient-based analysis.

		Number of patients, n (%)	Shrinkage rate (median [95%CI])	P value
SRS score	score 2	1 (5)	31.3 [-]	0.5439
	score 3	1 (5)	37,5 [-]	0.7948
	score 4	18 (90)	30.9 [0.29~27.3]	0.5287
Primary lesion	Pancreas	10 (20)	28.6 [9.82~47.4]	0.8494
	Rectum	5 (25)	13.7 [-30.3~57.7]	0.2056
	GI	2 (10)	41.0 [-162.3~244,3]	0.7055
	Others	3 (15)	36.5 [30.8~42.3]	0.7913
Ki67 labeling index	<10%	14 (70)	33.5 [22.1~44.9]	0.7415
	>10%	6 (30)	27.3 [-11.3~65.7]	
Therapeutic drug	^{90}Y and ^{177}Lu-DOTATOC	11 (55)	32.9 [14.0~51.9]	0.4704
	^{177}Lu-DOTATOC	9 (45)	30.0 [12.8~47.1]	
Previous treatment				
Cytotoxic agent	yes	5 (25)	48.6 [29.8~67.4]	0.0887
	no	15 (75)	25.9 [11.8~40.1]	
Targeted molecular therapy	yes	16 (80)	27.5 [13.6~41.8]	0.0726
	no	4 (20)	47.8 [28.7~66.9]	
Somatostatin analog	yes	11 (55)	31.3 [17.3~45.3]	0.6214
	no	9 (45)	31.9 [8.2~55.6]	
SSA maintenance treatment	yes	7 (35)	22.1 [-6.4~50.6]	0.4516
	no	13 (65)	36.7 [24.0~49.4]	
Tumor size before PRRT	<3.0cm	9 (45)	33.5 [17.1~49.8]	0.8494
	>3.0cm	11 (55)	30.1 [10.7~49.4]	

Abbreviations: NET, neuroendocrine tumor; SRS, somatostatin receptor scintigraphy; CI, confidence interval; GI, gastrointestinal tract; SSA, somatostatin analog; PRRT, peptide receptor radionuclide therapy
